# Factors Affecting Consumer Food Preferences: Food Taste and Depression-Based Evoked Emotional Expressions with the Use of Face Reading Technology

**DOI:** 10.1155/2019/2097415

**Published:** 2019-04-21

**Authors:** Elena Bartkiene, Vesta Steibliene, Virginija Adomaitiene, Grazina Juodeikiene, Darius Cernauskas, Vita Lele, Dovile Klupsaite, Daiva Zadeike, Laura Jarutiene, Raquel P. F. Guiné

**Affiliations:** ^1^Lithuanian University of Health Sciences, A. Mickeviciaus str. 9, LT-44307 Kaunas, Lithuania; ^2^Kaunas University of Technology, Radvilenu rd. 19, LT-50254 Kaunas, Lithuania; ^3^CI&DETS/CERNAS Research Centre, Polytechnic Institute of Viseu, Campus Plitécnico, Repeses, 3504-510 Viseu, Portugal

## Abstract

In this study, several factors (social status, age, gender, education, knowledge about healthy eating, and attitude to food) affecting consumer food choices (FC), including the relationship between the taste of food, FC, and depression, were analysed by using sensory traits and face reading technology. The first stage of the experimental scheme was the analysis of factors affecting consumer food preferences by using a questionnaire, while the second stage was evaluation of emotional expressions evoked by different food tastes in individuals with and without depressive disorders (DD), using the FaceReader 6 software. We show that gender is a significant factor for most emotional motivations, with a higher effect in females where there was an indication of increased cravings for sweets when feeling depressed. Age was a significant factor in the motivation to eat for positive feelings, while education had a significant influence on perceptions regarding healthy eating. Face reading technology was found to be sufficiently accurate to detect differences in facial expressions induced by different tastes of food, for groups with and without DD. In conclusion, many factors are of high importance in the analysis of food choices, and the results obtained using the FaceReader 6 technique are very promising for food-mood relation analysis. We suggest that mood has a strong link with the choice of food.

## 1. Introduction

The appropriate intake of nutrients has a major influence on public health [1] and is a major challenge for nutrition professionals when recommending balanced and/or healthy diets and affecting dietary change when necessary. It also affects the food choice of consumers. Many factors have an influence on food choice, which has led to the development of new food product technologies, as well as foods with new textures, tastes, and aroma characteristics to improve available food choices. Many people find it hard to change their dietary choices, which often occur impulsively and without deliberation; it is however unclear whether impulsive food choices can be experimentally created [2]. The major determinant of food choice is hunger, but if we have options what we choose to eat is not determined solely by physiological or nutritional needs. The consumers' gender, age, and education level, along with perception, emotional motivations, and selection of information sources about healthy eating should also be taken into account. According to the European Food Information Council (EUFIC), food induced emotions are very important, as food choice can also depend on our mood (EUFIC 2018). Mood is a complex human mental situation which fluctuates depending on several central and peripheral biological factors and other extraneous factors, including food. Essentially, good or bad moods are the result of certain chemicals influencing a neural response, and some foods have proved to be mood enhancers by affecting the release of desired neurotransmitters in the brain and also by relieving stress [3]. In humans, eating behaviour is complex and is affected by both moods and emotions; in addition, food consumption is important for mood-regulating behaviours. The interaction between mood, emotional state, and eating behaviours is varied, and it is hypothesised that individuals can regulate their emotions and moods by changing both food choices and quantities. A bidirectional link was suggested between good nutrition and psychological health, with evidence that individuals with a healthy diet are less likely to be depressed or develop depression [4]. On the other hand, depression can influence food choices via physiological processes that influence appetite or other behaviours that constrain or alter food availability [5].

The aim of this study was to evaluate the association between consumers' gender, age, and education level with their perception, emotional motivations, and knowledge about healthy eating. In addition, to evaluate a possible relationship between food choice and a person's mood, a study of emotions induced by different tastes of food in people with and without depressive disorder (DD) was performed.

## 2. Materials and Methods

### 2.1. Questionnaire Data Collection

This study involved 505 participants aged between 18 and 85 years, with 75.3% women and 24.7% men. Most participants (54.2%) had a university degree, 33.3% completed secondary school, and 12.5% finished primary school only. Demographic data of the sample studied is given in Table 1. To evaluate perceptions of healthy eating, sources of information about healthy diets, and emotional motivations associated with food choices and eating practices, a questionnaire was used [6].

### 2.2. Evaluation of Emotions Induced by the Different Tastes of Food in People with and without DD

**Figure 1:**
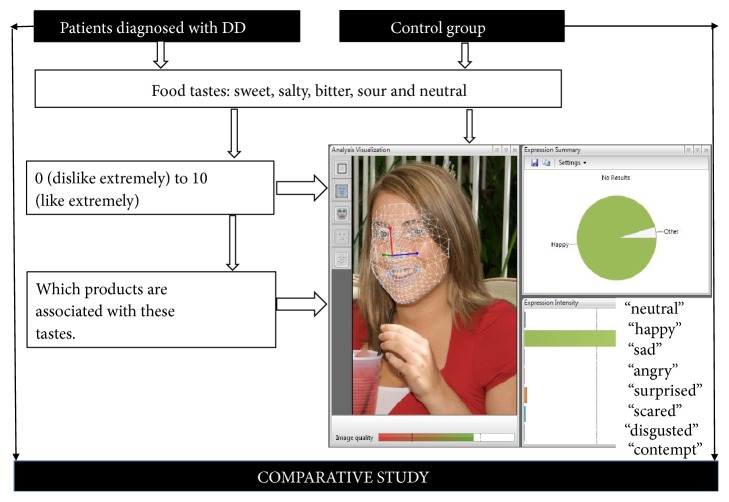
Principal scheme of the primary pilot comparative study of the food induced emotions of people with and without depressive disorder (DD).

To evaluate possible differences in food induced emotions in people with and without DD, the FaceReader technique (ver. 6; Noldus Information Technology, Wageningen, The Netherlands) was used. The principal scheme of the experiment is shown in Figure 1.

Two groups of subjects were invited to participate in the study: (I) patients diagnosed with DD and (II) a control group of subjects, not diagnosed with a mental disorder for at least a 1-year period. Their age varied between 18 and 55 years old. The severity of DD symptoms among patients was evaluated using a standard instrument, the Montgomery and Asberg Depression Rating Scale (MADRS) [7], at the Psychiatry Clinic of the Lithuanian University of Health Sciences (Kaunas, Lithuania).

The capture and analysis of the facial emotional expressions of patient and control group subjects, as well as emotional responses to different tastes of food, were carried out using FaceReader software in the morning (after 8 hours of fasting, but with no limit to water intake). In parallel, the acceptability of different food tastes was evaluated using a 10-point Likert scale from 0 (dislike extremely) to 10 (like extremely). Different food tastes (“sweet”, “salty”, “bitter”, “sour”, “neutral”) were presented by displaying cards showing the name of the particular food taste, and the tested individual was then asked to explain with which food product the presented taste is associated. The person was then asked to grade using the scale from 0 (dislike extremely) to 10 (like extremely) how much they liked the taste and/or product associated with the taste. No timer was used, to allow natural facial expressions. The whole procedure was filmed using a Microsoft LifeCam Studio webcam mounted on a laptop facing the participants, and Media Recorder (Noldus Information Technology, Wageningen, The Netherlands) software. Special care was taken to ensure good illumination of participant faces. The recordings, using a resolution of 1280×720 at 30 frames per second, were saved as AVI files and analysed frame by frame with FaceReader 6 software, scaling the 8 basic emotion patterns (neutral, happy, sad, angry, surprised, scared, disgusted, and contempt) to 1 (maximum intensity of the fitted model). In addition, the FaceReader also analysed the valence, which indicates whether the person's emotional status is positive or negative. ‘Happy' is the only positive emotion, while ‘Sad', ‘Angry', ‘Scared', and ‘Disgusted' are considered to be negative emotions. ‘Surprised' can be either positive or negative. The valence is calculated as the intensity of ‘Happy' minus the intensity of the negative emotion with the highest intensity. Valence scores ranged from -1 to 1.

For each food taste sample, the section of intentional facial expression (from the exact point at which the subject had finished raising their hand to give the signal until the subject started lowering their hand again) was extracted and used for statistical analysis. The FaceReader contains an image quality bar, which gives a good indication of how well the program is able to model the face depicted in the image. For the best image quality, the main attention was focused on camera position and illumination. For this reason, participants were asked to sit and look directly into the camera. For statistical analysis, the maximum values of facial expression patterns of the respective sections were used.

### 2.3. Statistical Analysis

Data analysis was performed using SPSS software from IBM, Inc. (version 24). The relationship between consumer gender, age, and education level with their perception, emotional motivations, and selection of information sources about healthy eating was evaluated using a descriptive statistics crosstabs test. The influence of the analysed factors was considered to be statistically significant when* p* ≤ 0.05. For the study of different tastes in food induced emotions in people with and without DD, 40 subjects suffering from DD and 40 control subjects were tested.

### 2.4. Ethical Approval

All ethical issues were verified when formulating and applying the questionnaire, which was approved by the Ethical Committee with reference no. BEC-MF-147. For the study of different tastes in food induced emotions in people with and without DD, approval to conduct the study was received from the Bioethics Committee (No. 04/2017). All subjects were informed about the study using a “personal information form”. Subjects were included in the study if they agreed to participate and signed an “informed consent form”. The study was conducted in accordance with the guidelines of Good Clinical Practice and the principles of the Declaration of Helsinki.

## 3. Results and Discussion

### 3.1. Relationship between Consumer Gender, Age, and Education Level and Their Perceptions, Emotional Motivations, and Selection of Information Sources Regarding Healthy Eating

**Figure 3.1:**
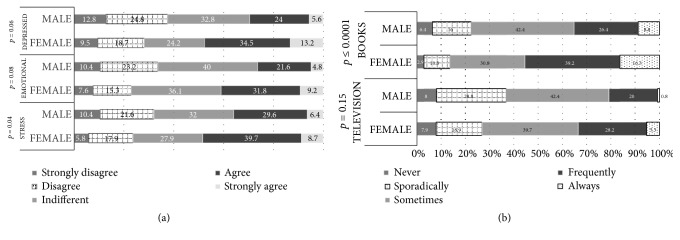
Influence of consumer gender on their (a) emotional motivations and (b) selection of information sources about healthy eating.

Perceptions of healthy eating can be considered to be one of the many factors influencing people's eating habits and choice of food. Scientists, dietitians, and the public agree that an optimal diet should be a primary focus of a healthy lifestyle [8, 9]. More data are needed on perceptions of healthy eating in general, on the influence of information from diverse sources such as food companies, and most importantly on the role of perceptions of healthy eating as a determinant of food choice [10]. Results regarding the influence of different factors (gender, age education level) on consumer perceptions, emotional motivations, and the selection of information sources about healthy eating are presented in Figures 3.1–4.

Evaluation of consumer perceptions about healthy eating showed that gender is not a significant factor; however, a significant influence was observed for gender on emotional motivations such as ‘food helps me cope with stress', ‘food serves as emotional consolation' and ‘I have more cravings for sweets when depressed' (*p* = 0.04,* p* = 0.08,* p* = 0.06, respectively) (Figure 2(a)).

When comparing female and male participants for the perception ‘food helps me cope with stress', 8.7% of females and 6.4% of males ‘strongly agree', while 39.7% of females and 29.6% of males state that they ‘agree'. Greater differences were found between female and male respondents when analysing the perceptions ‘food serves as emotional consolation' and ‘more craving for sweets when depressed', with ‘strongly agree' indicating for 9.2% and 13.2% of females, and 4.8% and 5.6% of males, respectively. Also, gender has a significant influence on the selection of sources of information about a healthy diet: according to questionnaire data, information ‘sporadically' obtained information from television (*p* = 0.015) and books (*p* ≤ 0.0001) was higher in males (16.0% and 28,8%) than in females (10.8 % and 18,9 %)); in contrast, females obtained information from books and television more ‘frequently' (39.2% and 28,2%) than males (26.4% and 20,0%) (Figure 2(b)).

There were a number of important relationships observed between stress, coping mechanisms, and lifestyle. Relationships between job stress and maladaptive or unhealthy coping behaviours were more clearly demonstrated in men than in women, particularly with respect to excessive consumption behaviour (food, cigarettes, and alcohol) and denial of feeling stress. Men reported significant associations between job stress, drinking status, and unhealthy eating patterns [11]. Some studies have shown that, compared to men, women are more aware of and attentive to their emotions and more likely to engage in concerted efforts to change them. Women show more awareness of their own emotions, and those of others, and pay more attention to them compared to men, on both self-report and performance-based measures [12]. Data from a representative survey of the Norwegian population showed that women considered health aspects and accordingly chose foods they consider to be healthy more often than men, when selecting foods for an everyday dinner [13].

**Figure 3:**
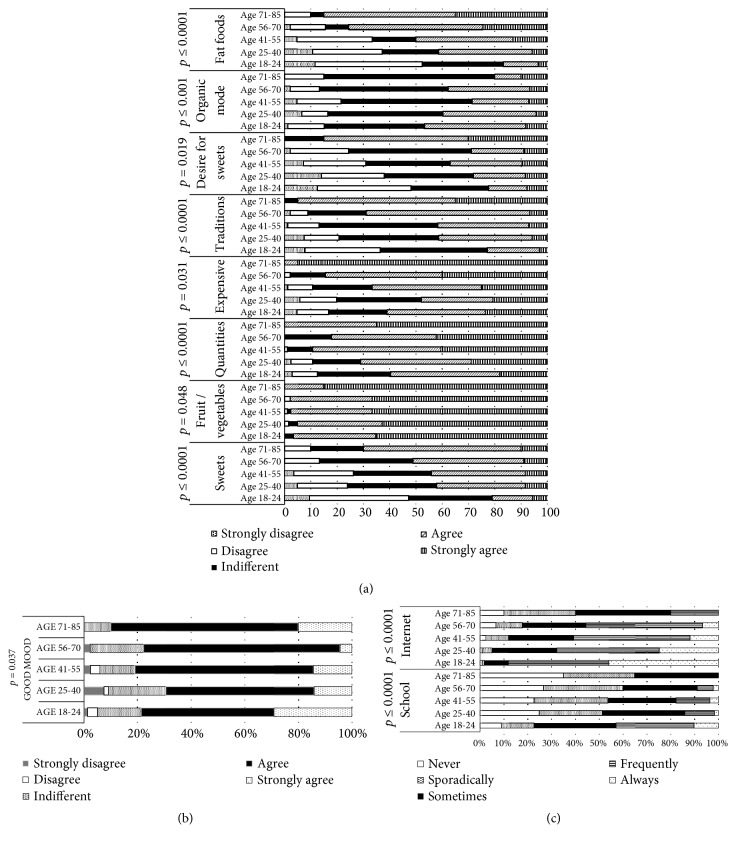
Influence of consumer age on their (a) perception, (b) emotional motivations, and (c) selection of information sources regarding healthy eating.

In our study, the age of respondents had a significant influence on the emotional motivation ‘food makes me feel good' (*p* = 0.037); however, other analysed emotional motivations such as ‘food helps to control weight' or ‘eating when feeling lonely' and ‘I eat more when there is nothing to do' were not influenced by age (Figure 3(b)). It has previously been shown that people usually change their eating behaviours when they perceive themselves to be stressed or are under persistent external interpersonal, financial, or other strains [14, 15].

Although approximately 20% of people do not change their eating behaviours during stressful periods, the majority do; approximately 40% or more increase and 40% or less decrease their caloric intake when stressed [16, 17]. The age of respondents was a significant factor in perceptions of healthy eating (from* p* ≤ 0.0001 to* p* = 0.048, Figure 3(a)), as well as in perceptions of some of the sources of information for selection of a healthy diet, such as school and the press (*p* ≤ 0.0001; Figure 3(c)). Generally, previous tests have shown that TV or Internet sources can influence food choices and even food intake and that food preferences in young people are acquired through learning processes, with these preferences having long-lasting effects (18-40) (Figure 3(c)).

Different foods induce different emotions, and consumers choose foods depending on their mood. Age was found to be a significant factor for the ‘food–mood' association of participants in this study. It is suggested that ‘comfort foods' have a high calorie content and tend to be associated with childhood and/or home cooking, often prepared in a simple or traditional style. They may have nostalgic or sentimental appeal, perhaps reminding us of home, family, and friends [18]. Older people are more likely to report positive emotions after having eaten their favourite comfort food, and people tend to focus more on positive emotions/situations as they age [19, 20]. Our obtained results are in agreement with this finding, as 90% of the participants in the age group of 71–85 years old indicated ‘agree' and ‘strongly agree' when ‘food–mood' relationships were analysed.

There may also be a neuropsychopharmacological aspect to ‘comfort foods', as eating palatable foods can lead to the release of trace amounts of mood-enhancing opiates. However, in the younger age groups, the ‘food–mood' relationship was indicated by a lower number of participants (in the group of 25–40 year olds, it was 21% lower, while in the other groups it was an average of 11% lower) (Figure 3(a)).

**Figure 4:**
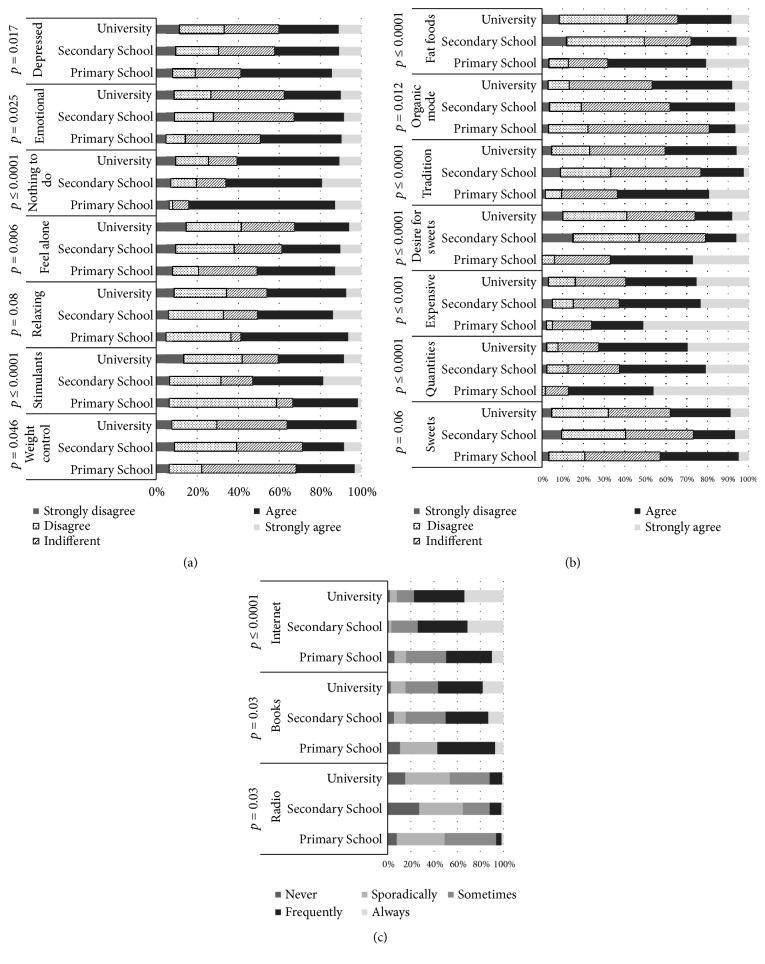
Influence of consumer education level on their (a) emotional motivations, (b) perception, and (c) selection of information sources about healthy eating.

Education has a significant influence on three out of the seven information sources (radio, press, and Internet (from* p* = 0.03 ≤ 0.0001, Figure 4(c)). 44.4% of participants with a primary school education level sometimes chose a radio as an information source. However, with increasing education level, the popularity of radio as information source was reduced, with only 37.5% and 38.3% of participants with an education level of secondary school and university indicating that they ‘choose sporadically' radio as a source, respectively. Books or magazines as an information source were “used sometimes” by 55.6% of participants with an education level of primary school, while this source was more popular in the participant group with higher education levels. In addition, only 10% of participants with a primary school education level indicated ‘always use Internet' as information source, while for respondents with an education level of secondary school and university this corresponded to 21% and 24%, respectively (Figure 4(c)).

Education was a significant factor (*p* = 0.012 ≤ 0.0001) on perceptions of healthy eating (Figure 4(b)). A high number of participants with an education level of primary school (38.1%) agreed that sweets are not a healthy food; however, most participants with a higher education level were indifferent on this point (on average 31.3%). In addition, about half of participants (46–51%) with an education level of primary school strongly agreed with ‘can eat everything in small quantities' and ‘healthy diet is not cheap', and 40–48% of these respondents agreed with the perceptions ‘tradition is very important to a healthy diet' or ‘never consume fat products' (Figure 4(b)). Compared with the higher education groups, it could be stated that people who have a lower education level have a lower tolerance of perceptions and believe popular healthy diet/eating claims more strongly.

For most of the analysed emotional motivations, participants' education level had a significant influence (*p* ≤ 0.05) (Figure 4(a)). However, 32–34% of participants with university education level agreed with the emotional motivation ‘usually eat foods that help control my weight', as well as with the perception ‘often consume foods that keep me awake'. Up to 52.4% of participants with an education level of primary school agreed with the emotional motivation ‘often consume food that helps'; this point was very popular with higher education groups (36.9% for secondary school and 38.7% for university). A high number of participants (38.1% and 71.4%) with an education level of primary school agreed that emotional motivations have an influence on eating habits. Most of the participants with a higher education level also agreed with the emotional motivation ‘eat more when I have nothing to do' (47.0% for secondary school and 50.0% for university). Also, higher numbers of primary school education participants (44.4%) were strongly influenced by the emotional motivation ‘have more cravings for sweets when I feel depressed', compared to participants with higher education levels (31.5% for secondary school and 29.2% for university). Previous studies have concluded that individuals with lower income were more likely to have lower levels of nutrition knowledge [21, 22], which is associated with lack of use of nutritional labels [23]. Similar effects have been observed for education levels: individuals with more education have reported a greater use of nutrition labels [24]. Several studies have shown that dietary quality seems to depend on socioeconomic variables such as occupation, income, and education [25]. Indeed, there is plenty of literature suggesting that, in developed countries, lower socioeconomic levels are traditionally characterized by unhealthier dietary habits as compared to upper socioeconomic levels [26].

### 3.2. Food Induced Emotions in People with and without Depressive Disorder

**Figure 5:**
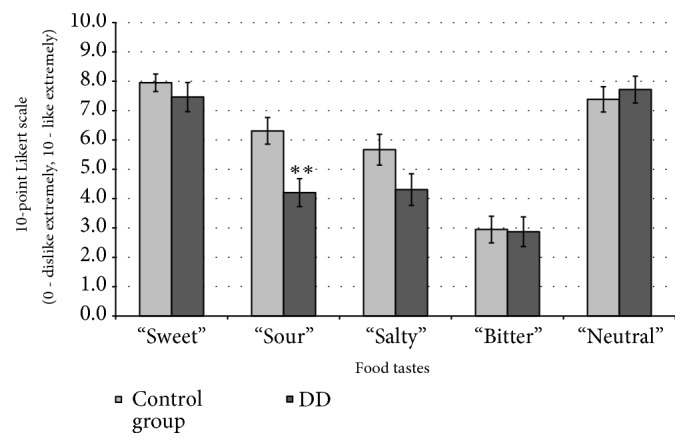
Different food tastes (sweet, salty, bitter, sour, and neutral) evaluated using a 10-point Likert scale (from 0: dislike extremely to 10: like extremely) in people with and without depressive disorder (DD). ∗∗: significant differences compared with control group (p ≤ 0.001).

**Figure 6:**
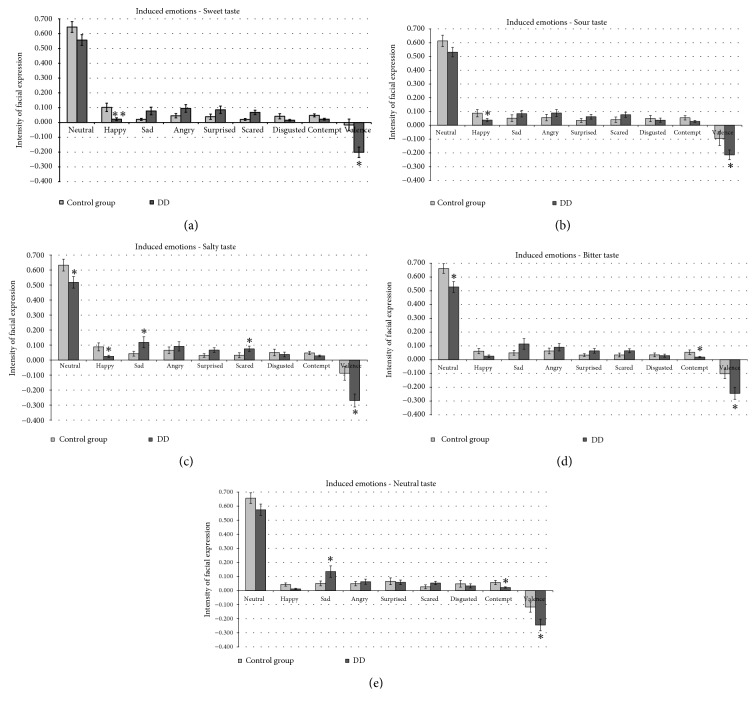
Emotions induced by different food tastes (sweet, salty, bitter, sour, and neutral) in people with and without depressive disorder (DD). ∗: significant differences compared to control group (p ≤ 0.05), ∗∗: significant differences compared to control group (p ≤ 0.001).

Food induced emotion results, presented as the mean values of analysed emotions and valence in the persons with DD (n=40) and in the control group (n=40), are shown in Figures 5 and 6.

*Hedonic Scale*. In persons with DD, “sour” taste showed 29.3% lower acceptability in the hedonic scale as compared to control group (p ≤ 0.001) (Figure 5). However, there was no other relationship between food taste and emotions when comparing the control group to people with DD.

*Sweet Taste*. Persons with DD expressed lower ‘‘happy” emotions for “sweet” taste compared to the healthy group (77.9% lower, p ≤ 0.001) (Figure 6(a)).

*Sour Taste*. Similar tendencies in the evaluation of “sour” tastes were obtained, with persons suffering from DD expressing 56.6% lower ‘‘happy” emotions compared to the healthy group (p ≤ 0.05) (Figure 6(b)).

*Salty Taste*. Similar to tendencies in “sweet” and “sour” tastes, in evaluation of “salty” tastes persons suffering from DD expressed 71.5 % lower ‘‘happy” emotions as compared to the healthy group (p ≤ 0.05). Furthermore, we found a statistically significant difference between people with DD and the control group in terms of the ‘‘neutral” emotion (18.1% lower, p ≤ 0.05). Opposite results in terms of the emotions ‘‘sad” and ‘‘scared” were obtained, as in all cases they were higher in persons with DD (64.0% and 57.1% higher, respectively, p ≤ 0.05) as compared to the healthy group (Figure 6(c)).

*Bitter Taste*. Lower ‘‘neutral” and ‘‘contempt” emotions were observed in persons with DD for “bitter” taste (20.2% and 64.6% lower, respectively, p ≤ 0.05) (Figure 6(d)).

*Neutral Taste*. Higher ‘‘sad” and lower ‘‘contempt” emotions were observed in persons with DD for “neutral” taste (62.3% higher and 61.3% lower, respectively, p ≤ 0.05) (Figure 6(e)).

In addition, valence results showed that the valence mean significantly correlated with the emotional state of persons with DD. People with DD showed an average decrease in valence mean for sweet, sour, salty, bitter, and neutral tastes of 91.9, 55.6, 67.4, 58.3, and 52.3%, respectively (p ≤ 0.05) compared to control group (Figure 6). This can be explained by the fact that people with DD are characterized by a low mood, accompanied by lowered self-esteem, and a loss of interest or pleasure in normally enjoyable activities (American Psychiatric Association, 2000). Our data are partly consistent with that of Ille et al. [27], who reported a response bias in patients with depressive disorder, who judged happy faces as less happy compared to healthy people.

According to the literature, food choice and emotions*‚* stress-related eating, eating comfort foods, and emotional eating have a direct relationship with negative mood states such as sadness, loneliness, and concern [28–30]. Beyond stress, which affects most of the population at some time, about 7% of the European population suffers from DD every year [31]. Only one investigation has directly tested the relationship between DD and food reward using behavioural paradigms [32]. Using a modified probabilistic incentive-learning task in which children were rewarded with candy (Skittles or M&Ms), depressive symptoms were found to be unrelated to response bias in food reward [33]. These findings complement results from sugar taste tests in which depressed and nondepressed adults responded similarly in terms of pleasure ratings for increasingly sweeter sucrose solutions [34–36]. These limited behavioural results do not suggest that depressive symptoms are associated with reduced reward functioning per se; however, in a sample of adolescents at risk for depression, high-risk youth demonstrated a reduced neural response to chocolate relative to low risk youth [37]. Overall, studies testing the relationship between depressive symptoms and food reward yield mixed findings, with behavioural results suggesting no relationship between depression and food reward and neuroimaging studies suggesting a decreased neural response to food in depression [38]. However, our results, obtained using* FaceReader* technique, are very promising and showed that emotions induced by different food tastes have a differing tendency when comparing healthy people to people suffering from DD. Our results also showed higher sensitivity as compared with evaluation using the hedonic scale, which can be influenced by previous emotions induced by a participant's past food use. These results are consistent with the concept that emotions have a uniquely important role in food consumption and affect eating responses along the entire process of ingestion [39]. Finally, we suggest that mood also has a relationship with the choice of food and that further studies are needed with a higher number of participants for evaluation of this process.

## 4. Conclusions

There are many relationships between consumer social status and perceptions of food. Gender is a significant factor in the emotional motivations of “food helps me cope with stress”, “for me, food serves as an emotional consolation”, and “I have more cravings for sweets when I am depressed”. Significant differences between female and male participants were observed as regards selection of the perception “for me, food serves as an emotional consolation” and “I have more cravings for sweets when I am depressed”. Participant age has a significant influence on the emotional motivation “food makes me feel good“, and education has a significant influence on perceptions regarding healthy eating. Overall, many factors are very important when food choices are being analysed, and personalised nutrition has become an important concept used to balance the diet of a population with different social statuses. Also, results obtained using face reading technology showed higher sensitivity than evaluations using a hedonic scale, which can be influenced by previous emotions of participants induced by past memories of foods. We suggest that mood also has a link to the choice of food. Finally, the Noldus* FaceReader* 6 software is very promising and sufficiently accurate to detect differences in facial emotion expressions induced by different tastes of food for different mood groups (with and without DD). However, more research is needed to determine how this technology performs in more complex testing procedures, in both simulated and “real life” environments.

## Figures and Tables

**Table 1:** Sociodemographic characterisation.

Sociodemographic data		Frequency	Percentage (%)
		(N)	

Gender	Female	380	75.3
	Male	125	24.7

Highest Level of Education	Primary School	63	12.5
	Secondary School	168	33.3
	University Degree	274	54.2

Total Number of Participants		505	

## Data Availability

The data of patients information used to support the findings of this study are restricted by the Bioethics Committee (No. 04/2017) in order to protect patient's privacy.
